# Protective Effects of Recombinant Kunitz-Domain 1 of Human Tissue Factor Pathway Inhibitor-2 Against 2-Chloroethyl Ethyl Sulfide Toxicity In Vitro

**Published:** 2007-07-10

**Authors:** Moonsuk S. Choi, Kalpana Parikh, Ashima Saxena, Nageswararao Chilukuri

**Affiliations:** Division of Biochemistry, Department of Molecular Pharmacology, Walter Reed Army Institute of Research, Silver Spring, MD 20910

## Abstract

**Objective:** Sulfur mustard is a well-known blistering chemical warfare agent that has been investigated for its toxicological mechanisms and an efficacious antidote. Since sulfur mustard injury involves dermal:epidermal separation, proteolytic enzymes were suspected to be involved for this separation and eventual blister development. Therefore, protease inhibitors could be of therapeutic utility against sulfur mustard injury. In this study, the effects of Kunitz-domain 1 of human tissue factor pathway inhibitor-2 were evaluated against the toxic effects of 2-chloroethyl ethyl sulfide, a surrogate agent of sulfur mustard. Tissue factor pathway inhibitor-2 is a 32-kDa serine protease inhibitor produced by a variety of cell types including human epidermal keratinocytes, fibroblasts, and endothelial cells. It consists of 3 Kunitz-domains and the first Kunitz-domain contains the putative P_1_ residue (arginine at position 24) responsible for protease inhibitory activity. **Methods:** Recombinant wild-type and R24Q mutant Kunitz-domain 1s were expressed in *Escherichia coli* and purified. The purified proteins were refolded, and their effects were tested in an in vitro human epidermal keratinocyte cell wounding assay. **Results:** Wild-type but not R24Q Kunitz-domain 1 inhibited the amidolytic activity of trypsin and plasmin. Wild-type Kunitz-domain1 was stable for 4 weeks at 42°C and for more than 8 weeks at room temperature. Wild-type Kunitz-domain 1 significantly improved wound healing of unexposed and 2-chloroethyl ethyl sulfide–exposed cells without influencing cell proliferation. Although R24Q Kunitz-domain 1 lacked trypsin and plasmin inhibitory activity, it promoted wound closure of untreated and 2-chloroethyl ethyl sulfide–treated cells but to a much lesser degree. **Conclusion:** These data suggest that wild-type Kunitz-domain 1 of human tissue factor pathway inhibitor-2 can be developed as a medical countermeasure against sulfur mustard cutaneous injury.

Sulfur mustard (HD, bis-(2-dichloroethyl) sulfide) is a chemical warfare agent that penetrates skin rapidly and causes erythema, edema, necrosis, and extensive blistering. Presently, there is no pretreatment or effective antidote for HD injury. Proteases are released and/or induced because of HD exposure and are suggested to be responsible for the formation of blisters.^1^–^3^ Therefore, protease inhibitors capable of inhibiting HD-induced and/or released proteases may offer protection against HD injury. Compound screening conducted by US Army Medical Research Institute of Chemical Defense revealed that topical application of serine protease inhibitors, namely, *N*-tosyl-l-lysine chloromethyl ketone and ethyl *p*-guinidinobenzoate hydrochloride, reduced HD toxicity in the mouse ear vesicant model. On the basis of these studies, it was suggested that serine protease inhibitors could be beneficial against HD toxicity in vivo.^4^ Recently, it was found that serine and matrix metalloprotease (MMP) inhibitors, namely aprotinin and ilomastat, have offered protection against HD-induced lung injury in rats.^5^,^6^

Tissue factor pathway inhibitor-2 (TFPI-2) is a member of the Kunitz-type family of serine protease inhibitors with strong homology to the classical tissue factor pathway inhibitor (TFPI-1).^7^ It is a 32-kDa broad-spectrum serine protease inhibitor and inhibits a variety of proteases including trypsin, plasmin, chymotrypsin, cathepsin G, plasma kallikrein, and factor VIIa–tissue factor complex with nanomolar affinity.^7^,^8^ TFPI-2 has also been reported to inhibit some MMPs either directly or indirectly. While the study conducted by Du et al failed to observe inhibition of activated MMP-1 by recombinant TFPI-2 and its binding to MMP-1, MMP-2, and MMP-9,^9^ some studies observed direct inhibition of MMP-1, MMP-2, MMP-9, and MMP-13.^10^,^11^ We reported the ability of TFPI-2 to negatively regulate MMPs including MMP-1, MMP-2, and MMP-9 by inhibiting their activation from proenzymes.^12^ A wide variety of cells, including human epidermal keratinocytes, dermal microvessels, and dermal fibroblasts, synthesize and secrete TFPI-2.^13^–^15^ Secreted TFPI-2 tightly binds to connective tissue matrix involving glycosaminoglycans.^16^,^17^ Structurally, TFPI-2 consists of 3 Kunitz-domains (KDs) and the first KD (KD1) contains putative P_1_ residue (arginine at position 24) that is responsible for the inhibition of trypsin, plasmin, and factor VIIa-tissue factor complex.^18^ Mutating the P_1_ residue from arginine (R) to glutamine (Q) (R24Q) caused significant loss of the inhibitor's activity toward trypsin, plasmin, and factor VIIa-tissue factor activity.^18^ Human recombinant TFPI-2 inhibited extracellular matrix destruction and invasion by cancer cells and tumor angiogenesis.^19^ In light of these properties, such as broad specificity and extracellular matrix localization, TFPI-2 has a possible role in extracellular matrix degradation and remodeling associated with physiological and pathological situations including cell migration, cell invasion, wound healing, angiogenesis, atherosclerosis, and tumor invasion and metastasis.

In this study, we tested the protective effects of human recombinant wild-type KD1 (wt-KD1) and R24Q mutant KD1 (R24Q-KD1) against the toxic effects of 2-chloroethyl ethyl sulfide (CEES), a surrogate agent of HD, in an in vitro human epidermal keratinocyte (HEK) cell wounding assay. Our results suggest that wt-KD1 and, to a significantly lesser extent, R24Q-KD1 promoted wound closure of unexposed and CEES-exposed HEK, suggesting beneficial effects for wt-KD1 against HD injury.

## MATERIALS AND METHODS

### Materials

Expression vector, pET15b, and *E coli* strain BL21(DE3) were purchased from Novagen Inc (Madison, WI). Quick Ligation Kit and restriction endonucleases, Nde1 and BamH1, were purchased from New England Biolabs (Beverly, MA). DNA purification kits were obtained from Qiagen Inc (Valencia, CA). Thrombin CleanCleave Kit, chromogenic substrate H-D-Val-Leu-Lys-*p*-nitroanilide (S2251), human plasmin, bovine trypsin, and all other chemicals were obtained from Sigma Chemical Co (St Louis, MO). QuikChange XL site-directed mutagenesis kit was purchased from Stratagene (La Jolla, CA). Amicon Ultra-15 centrifugal filter devices were purchased from Millipore Corp (Bedford, MA). HEK was obtained from ATCC (Manassas, VA). Defined keratinocyte-SFM, growth supplements, and trypsin-EDTA were purchased from Life Technologies Inc (Gaithersburg, MD). Pfu DNA polymerase, calf intestinal alkaline phosphatase, and CellTiter 96 AQ_ueous_ One Solution Cell Proliferation Assay kit were purchased from Promega Corp (Madison, WI). Nickel–Sepharose high-performance resin for His-Trap column was obtained from Amersham Biosciences Corp (Piscataway, NJ). Coomassie Blue (Biosafe), acrylamide and bisacrylamide, silver staining, and protein determination kits were obtained from Bio-Rad Laboratories (Richmond, CA). Recombinant human TFPI-2 expressed from mammalian cells and anti-human TFPI-2 IgG were generously provided by Dr Walter Kisiel (University of New Mexico Health Sciences Center, Albuquerque, NM).

### Expression and purification of wt-KD1 and R24Q-KD1

DNA encoding the wt-KD1 of human TFPI-2 was cloned into pET15b expression plasmid according to standard procedures. With this expression system, KD1 was made as a fusion protein containing 6 X histidine tag at its amino terminus, which could be removed by incubation with thrombin. To make a point mutation on KD1 (R24Q), site-directed mutagenesis was performed using QuickChange XL site-directed mutagenesis kit according to the manufacturer's instruction. The recombinant construct was examined for in-frame orientation and integrity by nucleic acid sequencing. The KD1 fusion proteins were expressed in *E coli* grown in LB broth containing 100 μg/mL of ampicillin and induced at 37°C with 1 mM isopropyl-thiogalactopyranoside (IPTG) at mid-log phase (A_600_ = 0.6–0.7) for 2 hours.

Induced cells were harvested and lysed by French Pressing in 30 mM Tris-HCl (pH 8.0) containing 1 mM EDTA. Inclusion bodies were collected by subjecting the lysate to centrifugation at 17000 × *g* for 30 minutes at 4°C and washed once with the same buffer. The inclusion bodies were solubilized overnight in PBS containing 6 M guanidine HCl and centrifuged at 12000 × *g* for 30 minutes at 4°C. Supernatant was collected and filtered through 0.2 micron pore size filters, and then the filtrate was loaded onto a His-Trap column. The column was washed with PBS containing 6 M guanidine HCl (equilibration buffer), followed by washing with equilibration buffer containing 25 mM imidazole. wt-KD1 fusion protein was eluted from the column in equilibration buffer containing 500 mM imidazole.

His-trap column eluted wt-KD1 was not active against trypsin and plasmin. To recover the enzyme inhibitory activity for His-trap column eluted wt-KD1 fusion protein, it was reduced and refolded as described.^20^,^21^ Refolded wt-KD1 fusion protein was then filtered through 0.2 micron pore size filters and subjected to fast protein liquid chromatography (FPLC), using HiTrap Q anion exchange column. Protein was eluted from the column using a linear 0–1 M NaCl gradient. Column fractions were analyzed by SDS-PAGE followed by Western blotting and/or silver staining to identify wt-KD1 fusion protein. Fractions containing pure wt-KD1 fusion protein were pooled and digested with thrombin using Thrombin CleanCleave Kit following the manufacturer's protocol. Complete digestion of wt-KD1 fusion protein by thrombin was confirmed by SDS-PAGE. Thrombin-cleaved wt-KD1 was applied onto Amicon Ultra-15 centrifugal filter device for removing His_6_ peptide and for concentration of the sample. Each batch of the pure wt-KD1 was characterized with respect to protein concentration, purity, and inhibition kinetics as previously described.^8^

R24Q mutant KD1 was also expressed and purified by His-trap column and FPLC anion-exchange chromatography as described above for wt-KD1.

### Trypsin and plasmin inhibition assays

The inhibitory activities of TFPI-2, wt-KD1, and R24Q-KD1 against trypsin and plasmin were determined as previously described.^8^ Trypsin (0.1 nM) and plasmin (0.2 mU) were each incubated with various concentrations of TFPI-2, wt-KD1, and R24Q-KD1 for 15 minutes at room temperature. Chromogenic substrate (S-2251, 0.08 mM) was then added, and residual amidolytic activity was measured at 405 nm using Spectramaxplus microplate reader (Molecular Devices). Full-length TFPI-2 expressed in mammalian cells was included as a positive control.

### In vitro wound healing assay

The in vitro wound healing assay using HEK was performed as described.^22^ HEK cells were seeded in 12-well tissue culture plates (2 × 10^5^ cells/per well) and grown in defined keratinocyte-serum-free medium containing growth supplements. Twenty-four hours after seeding, a linear wound was made by scraping HEK monolayer with a pipette tip, followed by extensive washing with the growth medium to remove cellular debris. The wt-KD1 (1, 10, 20, 50, or 100 nM) or R24Q-KD1 (1, 10, 20, 50, or 100 nM) were added immediately after washing, and wound closure was monitored for 48 hours. Cell images were obtained using an inverted microscope (Olympus 1 × 71) attached with a camera (Olympus DP12).

In some experiments, HEK monolayers were exposed to 0 to 500 μM CEES for 24 hours. Then a linear wound was made and wound closure was monitored and recorded as described above. To investigate the effect of wt- and R24Q-KD1s, 125 μM CEES-exposed and wounded HEK were incubated with increasing concentrations of these 2 proteins (10, 20, 50, or 100 nM), and monitored for wound closure as described above.

### Cell proliferation assay

HEK proliferation rate was measured by using a CellTiter 96 AQueous one-solution cell proliferation assay. HEK cells were plated in 96-well plate at a density of 3000 cells per well. Next day, the cells were treated with wt-KD1 (1, 10, 20, 50, or 100 nM) or R24Q-KD1 (1, 10, 20, 50, or 100 nM) for 48 hours. Then, 20 μL of CellTiter 96 AQ_ueous_ one-solution was added to each well. After 3 hours of incubation, absorbance at 490 nm was measured using Spectramaxplus microplate reader. All assays were done in triplicates, and average and the standard deviation were shown.

In some experiments, HEK were first exposed with 125 μM CEES for 24 hours and then treated with wt-KD1 (1, 10, 20, 50, or 100 nM), or R24Q-KD1 (1, 10, 20, 50, or 100 nM) for 48 hours and cell proliferation was determined as described above.

### Western blotting

Proteins were boiled for 5 minutes, separated by SDS-PAGE using 15% polyacrylamide gels, and elctroblotted onto nitrocellulose membrane.^23^ After elctroblotting, the membranes were blocked with 3% nonfat dry milk in 10 mM Tris-HCl with 150 mM NaCl (pH 7.4) and 0.1% Tween-20 (TTBS) for 2 hours at room temperature. The membranes were then incubated overnight at 4°C with anti-TFPI-2 antibody (diluted 1:3000 in TTBS with 1% BSA). After several washes, the membranes were incubated for 1 hour with peroxidase-conjugated secondary antibody (diluted 1:3000 in TTBS containing 1% BSA). After several washes in TTBS, the immunoreactive proteins were identified by using an enhanced chemiluminescence reagent system according to the manufacturer's instructions.

### Other methods

SDS-PAGE was performed using 15% or 18% mini-slab gels.^24^ Proteins were identified by staining with Coomassie blue. Protein concentration was determined by the dye binding method with a Bio-Rad protein assay kit according to the manufacturer's instructions. Silver staining was performed using the Bio-Rad silver stain kit following the manufacturer's instructions.

## RESULTS

### Expression, purification, and refolding of wt-KD1

**Figure 1:**
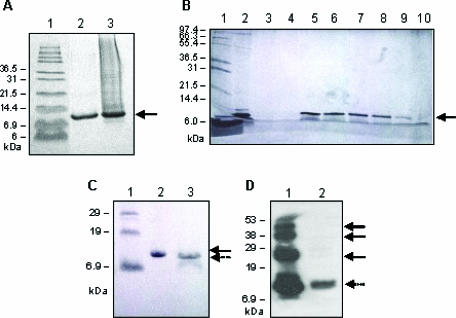
Prokaryotic expression and purification of wt-KD1: Panel A: SDS-PAGE of His-affinity column purified wt-KD1. Lanes are (1) molecular weight markers; (2) column purified wt-KD1 and (3) inclusion body extract. The arrow points to the location of wt-KD1. Panel B: SDS-PAGE of FPLC fractions. FPLC fractions were processed by SDS-PAGE, using 15% polyacrylamide gel, and proteins were identified by silver staining. Lanes are (1) molecular size markers; (2) wt-KD1 purified with His-affinity column; and (3–10) FPLC fractions from A6–A13. Fractions A8–A12 (lanes 5–9) contained the pure wt-KD1 fusion protein. Panel C: SDS-PAGE of wt-KD1 without His-tag. Lanes are (1) molecular size marker; (2) wt-KD1 with His-tag (solid arrow); and (3) wt-KD1 without His-tag (dashed arrow). Panel D: Molecular composition of refolded wt-KD1. Proteins were fractionated using 15% polyacrylamide gels and identified by Western blotting using anti-TFPI-2 antibody. Lanes are (1) wt-KD1 with His-tag and (2) wt-KD1 without His-tag. Wt-KD1 with His-tag contains both monomers (dashed arrow) and multimers (solid arrows) where as wt-KD1 without His-tag contains monomers only.

Wt-KD1 protein was expressed in *E coli* grown in LB containing 100 μg/mL of ampicillin. Induction at 37°C with 1 mM IPTG at mid-log phase for 2 hours showed the highest amount of wt-KD1 expression (Figure 1A, lane 3). These conditions were adopted for the large-scale expression of both wt- and R24Q-KD1s.

The expressed wt-KD1 went into inclusion bodies, suggesting that the protein was insoluble and biologically inactive (Figure 1A, lane 3). Inclusion bodies were solubilized with 6 M guanidine HCl and His-affinity chromatography was performed to purify the wt-KD1. Refolding of His-affinity column purified wt-KD1 was accomplished by subjecting the protein to reduction with 50 mM dithiothreitol and then dialysis in a buffer containing 2 M urea, 0.3 M NaCl, 2.5 mM reduced glutathione, 0.5 mM oxidized glutathione, and 0.2 M arginine for 96 hours.^20^ SDS-PAGE of the refolded wt-KD1 suggests that it was relatively a pure protein with a molecular size of 10.6 kDa (Figure 1A, lane 2). This analysis also revealed that the purified wt-KD1 sample contained few minor contaminating proteins, specifically with molecular size more than 55 kDa (Figure 1A, lane 2). An average of 3.5 mg of wt-KD1 was obtained from 1 L of the IPTG-induced LB broth.

To remove the high-molecular-weight contaminating proteins, wt-KD1 was then subjected to FPLC using HiTrap Q HP column. Wt-KD1 binds to HiTrap Q HP resin and was eluted from the column with a salt concentration ranging from 0.2 M to 0.4 M. FPLC column fractions were assessed for wt-KD1 by SDS-PAGE followed by silver staining (Figure 1B). Column fractions 8–12 contained the pure wt-KD1, suggesting that high-molecular-weight contaminants were removed by FPLC anion exchange chromatography (Figure 1B, lanes 5–9).

To remove the His-tag from the refolded wt-KD1, FPLC fractions containing wt-KD1 were pooled, concentrated, and incubated with thrombin. SDS-PAGE of the thrombin digest reveals that the molecular size of wt-KD1 without the His-tag was 8.7 kDa (Figure 1C, lane 3) as compared with 10.6 kDa for the wt-KD1 with His-tag (Figure 1C, lane 2). Complete removal of His-tag from refolded wt-KD1 fusion protein was also confirmed by Western blotting with anti 6 X histidine antibodies. The antibody reacted with refolded wt-KD1 before incubation with thrombin but not after incubation (data not shown). Collectively, the data show that His-tag was completely removed from refolded wt-KD1.

It was shown that refolding of recombinant wt-KD1 fusion protein yields monomeric and multimeric forms.^25^ Therefore, we analyzed the molecular composition of recombinant wt-KD1 by SDS-PAGE and Western blotting with anti-TFPI-2 antibodies (Figure 1D). Wt-KD1 before the removal of His-tag contained both monomeric and multimeric forms confirming the earlier observations (Figure 1D, lane 1). In contrast, His-tag–removed wt-KD1 was homogenously monomeric (Figure 1D, lane 2). These results suggest that the recombinant wt-KD1 used in the present study was monomeric form.

### Expression, purification, and refolding of R24Q-KD1

Methods described above for the expression, purification, and refolding of wt-KD1 were used for obtaining recombinant R24Q mutant KD1. SDS-PAGE confirmed that R24Q-KD1 was a pure protein with a molecular size similar to that of the wt-KD1 (data not shown).

### Enzyme inhibition of wt-KD1 and R24Q-KD1

**Figure 2:**
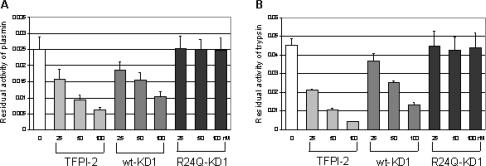
Inhibition on plasmin (A) and trypsin (B) by TFPI-2 (gray bars), wt-KD1 (dark grey bars), and R24Q-KD1 (black bars). Increasing concentrations of inhibitors were incubated with plasmin and trypsin as described under the section “Materials and Methods.” The residual amidolytic activity was represented as OD_405_/per minute. All values were the mean ± SD of triplicate samples.

In these studies, the ability of wt-KD1 and R24Q mutant KD1 to inhibit the amidolytic activities of bovine trypsin and human plasmin were compared (Figure 2). Full-length TFPI-2 expressed in mammalian cells was included as a positive control. Wt-KD1 inhibited the amidolytic activities of both trypsin and plasmin in a dose-dependent manner, suggesting that refolded wt-KD1 was biologically active (Figure 2A and 2B, dark gray bars). In contrast, R24Q-KD1 lacked inhibitory activity toward both trypsin and plasmin, suggesting the importance of arginine at position 24 for the inhibitor activity (Figure 2A and 2B, black bars). These results confirm the earlier observations that showed lack of enzyme inhibitory activity for R24Q-TFPI-2 against trypsin, plasmin, and factor VIIa-tissue factor complex.^18^

The inhibitory activities of wt-KD1 toward plasmin and trypsin were compared with those for the full-length mammalian TFPI-2. On a molar basis, wt-KD1 (Figure 2A and 2B, dark gray bars) was 40–50% less active than the full-length TFPI-2 (Figure 2A and 2B, gray bars). This observation contradicts an earlier study that showed that the trypsin and plasmin inhibitory activities for refolded wt-KD1 were stronger than those for full-length TFPI-2.^25^ Reason(s) for the discrepancy between our data and the data reported earlier by Chand et al^25^ may be due to the structural differences between the 2 proteins. Recombinant wt-KD1 produced in the previous study contains 10 X histidine tag whereas the wt-KD1 produced in the current study was without His-tag. Consequently, the molecular size of wt-KD1 in the earlier study was 12–14 kDa whereas it was 8.7 kDa for the wt-KD1 produced in the current study.

### Thermal stability of wt-KD1

**Figure 3:**
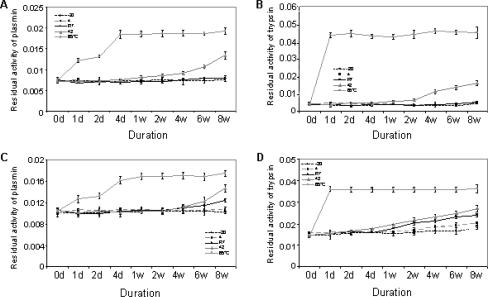
Thermal stability of wt-KD1. Wt-KD1 (panels A and B) and mammalian full-length TFPI-2 (panels C and D) were incubated at different temperatures for varying periods of time and their ability to inhibit amidolytic activities of plasmin (panels A and C) and trypsin (panels B and D) were determined. All values were the mean ± SD of triplicate samples.

In these studies, thermal stability of wt-KD1 was assessed and compared to that of TFPI-2 over an 8-week period (Figure 3). Recombinant proteins were incubated at different degrees of temperature, and their ability to inhibit plasmin and trypsin amidolytic activities were analyzed. At 65°C, the inhibitory activity of wt-KD1 toward plasmin was completely lost in 4 days (Figure 3A) whereas trypsin inhibitory activity was lost in 1 day (Figure 3B). Wt-KD1 was thermally stable for 4 weeks at 42°C and for more than 8 weeks at room temperature (Figure 3A and 3B). Similar results were observed for TFPI-2 (Figure 3C and 3D). These results suggest that the refolded wt-KD1 is stable for extended periods of incubation.

### Effect of wt- and R24Q-KD1s on the wound closure of HEK in vitro

**Figure 4:**
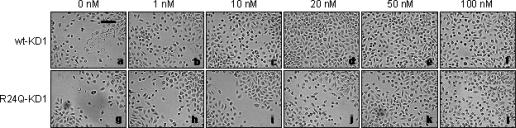
Effect of wt-KD1 and R24Q-KD1 on HEK wound healing. HEK cultures were wounded as described under the section “Materials and Methods” and incubated with either 0, 1, 10, 20, 50, and 100 nM of wt-KD1 (upper panels a–f, respectively) or 0, 1, 10, 20, 50, and 100 nM of R24Q-KD1 (lower panels g–l, respectively). Scale bar, 120 μm. The experiment repeated 3 times and similar results were observed.

We analyzed the effect of wt and R24Q-KD1s on the wound healing of HEK in an in vitro wound healing assay where the cells were wounded, treated with increasing concentrations (1–100 nM) of wt-KD1 or R24Q-KD1, and monitored for the wound closure by migrating cells for 48 hours. As shown in Figure 4, wound closure was faster for HEK treated with wt-KD1 but not with R24Q-KD1. At the end of 48 hours, wound closure was not observed for untreated HEK whereas it was complete for HEK treated with 20 nM or higher concentrations of wt-KD1 (Figure 4, upper panels) and to a significantly lesser extent with similar concentrations of R24Q-KD1 (Figure 4, lower panels).

### Effect of wt and R24Q-KD1s on the wound closure of HEK exposed to CEES

**Figure 5:**
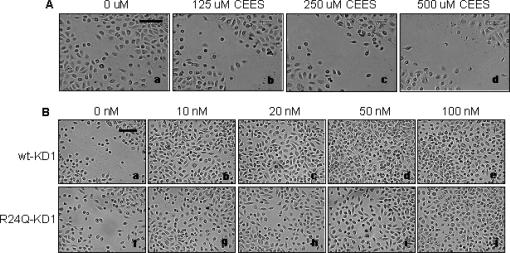
Effect of wt-KD1 and R24Q-KD1 on the wound closure of HEK exposed to CEES. A: HEK cultures were exposed to 0 μM (panel a), 125 μM (panel b), 250 μM (panel c), and 500 μM (panel d) of CEES for 24 hours, and wound closure was recorded after 48 hours. B: The CEES (125 μM) exposed HEK were wounded and then treated with 10, 20, 50, or 100 nM of wt-KD1 (panels b–e, respectively), or 10, 20, 50, or 100 nM of R24Q-KD1 (panels g–j, respectively), and wound closure was recorded after 48 hours. CEES-exposed HEK without any treatment represents as a control (panels a and f). Scale bar, 120 μm. The experiment was repeated 3 times and similar observations were made.

We also analyzed the effect of wt- and R24Q-KD1s on the wound healing of HEK exposed to CEES by the in vitro wound healing assay. First, the effect of increasing concentrations of CEES on HEK wound closure was studied. As shown in Figure 5A, CEES inhibited the wound closure of HEK in a dose-dependent manner. Wound gap was greater as the concentration of CEES was increased from 125 to 250 μM and from 250 to 500 μM.

Next, the effect of wt- and R24Q-KD1s on wound closure of 125 μM CEES-exposed HEK was investigated. In this experiment, confluent HEK cultures were first exposed with 125 μM of CEES for 24 hours and then wounded. Following wounding, HEK were incubated with increasing concentrations of wt and R24Q-KD1s (10–100 nM) and wound closure was monitored for 48 hours. As shown in Figure 5B, both wt (panels b–e) and R24Q-KD1 (panels g–j) promoted wound closure of CEES-exposed HEK in a dose-dependent manner. Nevertheless, the effect of wt-KD1 was much greater than that of R24Q-KD1 on a molar basis.

### Effect of wt-KD1 and R24Q-KD1 on proliferation of HEK

**Figure 6:**
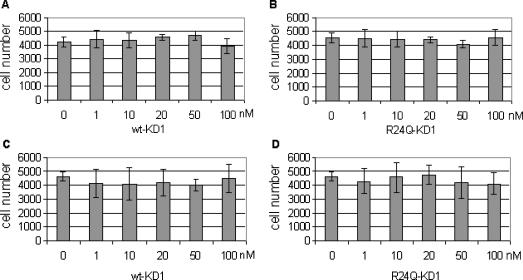
Effect of wt-KD1 and R24Q-KD1 on HEK proliferation. The cell proliferation of HEK were measured as described under the section “Materials and Methods” and incubated with either 0, 1, 10, 20, 50, and 100 nM of wt-KD1 (panel A) and 0, 1, 10, 20, 50, and 100 nM of R24Q-KD1 (panel B) for 24 hours and cell proliferation was measured with A_490_ after 48 hours. The CEES (125 μM) exposed HEK were treated with 0, 1, 10, 20, 50, or 100 nM of wt-KD1 (panel C), or 0, 1, 10, 20, 50, or 100 nM of R24Q-KD1 (panel D), and cell proliferation was measured with A_490_ after 48 hours. The cell number was calculated from absorbance at 490 nm. All values were the mean ± SD of triplicate samples.

To address the mechanism of enhanced wound closure of unexposed and CEES-exposed HEK by wt-KD1 and to a lesser extent by R24Q-KD1, their effects on HEK proliferation was investigated. As shown in Figure 6, both wt-KD1 (Figure 6A and 6C) and R24Q-KD1 (Figure 6B and 6D) have no influence on the proliferation of unexposed (Figure 6A and 6B) as well as 125 μM CEES-exposed HEK (Figure 6C and 6D) during a period of 48 hours. These results suggest that the wound closure effects of wt- and R24Q-KD1s are independent of HEK proliferation.

## DISCUSSION

In the present study, recombinant wt-KD1 was produced and tested for its efficacy in improving wound healing of CEES-injured HEK in an in vitro wound healing model. In this model, a linear wound was created by scraping cells and monitored for closure by migrating cells from the edge of the wound. Substances that help closing this artificial wound faster are considered to positively influence wound healing in vivo. Therefore, this assay may be considered as a prescreening model to select for substances/drugs with potential to be beneficial in the treatment of chemical and burn wounds in vivo. In this study, we used CEES, a surrogate agent of HD, to create artificial wounds and show that recombinant wt-KD1 enhances wound closure and that this protein may be beneficial against HD injury in vivo.

It has been suggested that proteases are necessary for the progression of HD-induced wounds and that protease inhibitors may offer protection. It was shown that certain synthetic serine protease inhibitors, namely TLCK, and compound 1579 suppressed HD-increased interleukin-8 in HEK as well as reduced HD toxicity in a mouse ear vesicant model.^1^–^6^ On the basis of these studies, we predicted that naturally occurring, stable, small molecular size, and broad-spectrum serine protease inhibitors could be beneficial in the treatment of HD wounds. Wt-KD1 of human TFPI-2 is an ideal candidate that has all the above-mentioned characteristics. It is (1) a 8.7-kDa protein, (2) produced in *E coli* in milligram quantities, (3) easily refolded so that the recombinant protein is biologically active, (4) stable at room temperature for nearly 8 weeks and for 4 weeks at 42°C, (5) exhibits inhibitory activity against trypsin, plasmin, and factor VIIa-tissue factor complex, and (6) its R24Q mutant KD1 lacking enzyme inhibitory activity provides a suitable control to understand the role of serine protease activity in HD toxicity. Therefore, we produced recombinant wt-KD1 and R24Q-KD1 and tested their beneficial activity against HD injury in the in vitro wound healing assay.

Our results provide evidence that wt-KD1 significantly promotes wound closure of unexposed and CEES-exposed HEK, suggesting that this protein is of therapeutic interest against HD injury. Surprisingly, R24Q-KD1 lacking trypsin, plasmin, and factor VIIa-tissue factor inhibitory activity also demonstrated wound-healing-promoting activity, suggesting that wound closure of unexposed and CEES-exposed HEK is regulated not only by serine protease inhibitory activity but also by other activities/mechanisms. Apparently, some of these activities are contained within R24Q-KD1 also. For example, Kamei et al^18^ found that R24Q-KD1, while lacking trypsin and plasmin activities, acquired strong inhibitory activity toward factor Xa. Therefore, R24Q-KD1 may have acquired the ability to influence certain proteases, as yet unidentified, which may play a role in the wound closure of HEK.

In summary, our results provide evidence that wt-KD1 of TFPI-2 could be beneficial against HD injury. Its biochemical and physical characteristics, such as small molecular size, broad specificity, and long-term stability at physiological temperatures and above makes it an attractive candidate as a medical countermeasure against HD injury.
